# Fluoxetine in Progressive Multiple Sclerosis (FLUOX-PMS): study protocol for a randomized controlled trial

**DOI:** 10.1186/1745-6215-15-37

**Published:** 2014-01-25

**Authors:** Melissa Cambron, Jop Mostert, Patrick Haentjens, Marie D’Hooghe, Guy Nagels, Barbara Willekens, Dorothea Heersema, Jan Debruyne, Wim Van Hecke, Luc Algoed, Nina De Klippel, Erwin Fosselle, Guy Laureys, Henri Merckx, Bart Van Wijmeersch, Ludo Vanopdenbosch, Wim Verhagen, Raymond Hupperts, Gerald Hengstman, Veronique Michiels, Annick Van Merhaegen-Wieleman, Jacques De Keyser

**Affiliations:** 1 Department of Neurology, University Hospital Brussel, Center for Neurosciences Vrije Universiteit Brussel (VUB) UZ Brussel, Laarbeeklaan 101, 1090 Brussels, Belgium; 2 Department of Neurology, Rijnstate Hospital, Arnhem, The Netherlands; 3 Centre for Outcomes Research and Laboratory for Experimental Surgery, Universitair Ziekenhuis Brussel, Vrije Universiteit Brussel, Laarbeeklaan 101, B 1090 Brussels, Belgium; 4 Department of Neurology, National MS Center Melsbroek, Brussels, Belgium; 5 Department of Neurology, Antwerp University Hospital, Antwerp, Belgium; 6 Department of Neurology, University Medical Center Groningen, University of Groningen, Groningen, The Netherlands; 7 Department of Neurology, University Hospital Ghent, Ghent, Belgium; 8 Icometrix, Leuven, Belgium; 9 Department of Neurology, AZ Maria Middelares, Ghent, Belgium; 10 Department of Neurology, Jessa Hospital, Hasselt, Belgium; 11 Department of Neurology, ASZ Aalst, Aalst, Belgium; 12 Department of Neurology, Maria Hospital, Halle, Belgium; 13 Department of Neurology, H.-Hartziekenhuis, Menen, Belgium; 14 Department of Neurology, MS centre, Overpelt, Belgium; 15 Department of Neurology, AZ St. Jan, Brugge, Belgium; 16 Department of Neurology, Canisius Wilhelmina Hospital, Nijmegen, The Netherlands; 17 Department of Neurology, Orbis Medisch Centrum, Sittard, The Netherlands; 18 Department of Neurology, Catharina Ziekenhuis, Eindhoven, The Netherlands

**Keywords:** Multiple sclerosis, Primary progressive, Secondary progressive, Clinical trial, Fluoxetine, Neuroprotection

## Abstract

**Background:**

Currently available disease-modifying treatments acting by modifying the immune response are ineffective in progressive multiple sclerosis (MS), which is caused by a widespread axonal degeneration. Mechanisms suspected to be involved in this widespread axonal degeneration are reduced axonal energy metabolism, axonal glutamate toxicity, and reduced cerebral blood flow. Fluoxetine might theoretically reduce axonal degeneration in MS because it stimulates energy metabolism through enhancing glycogenolysis, stimulates the production of brain-derived neurotrophic factor, and dilates cerebral arterioles. The current document presents the protocol of a clinical trial to test the hypothesis that fluoxetine slows down the progressive phase of MS.

**Methods/Design:**

The FLUOX-PMS trial is a multi-center, randomized, controlled and double-blind clinical study. A total of 120 patients with the diagnosis of either secondary or primary progressive MS will be treated either by fluoxetine (40 mg daily) or placebo for a total period of 108 weeks. The primary endpoint is the time to confirmed disease progression defined as either at least a 20% increase in the timed 25-Foot Walk or at least a 20% increase in the 9-Hole Peg Test. Secondary endpoints include the Hauser ambulation index, cognitive changes, fatigue, magnetic resonance imaging of the brain, and in a small subgroup optical coherence tomography.

**Discussion:**

The FLUOX-PMS trial will gives us information as to whether fluoxetine has neuroprotective effects in patients with progressive MS.

**Trial Registration:**

Eudra-CT: 2011-003775-11

## Background

Multiple sclerosis (MS) is a chronic inflammatory and degenerative disease and is considered the most important non-traumatic cause of neurological disability in young adults. Despite many decades of intensive research, the cause of MS has remained elusive, and many aspects of the pathogenesis are not understood. The disease appears to precipitate in genetically susceptible individuals, very likely as a result of an environmental trigger. An infectious component has long been suspected, but no specific transmissible agent has so far been linked convincingly to MS [1].

The disease course of MS is heterogeneous. A majority of patients initially has bouts of neurological deficit (relapses) followed by (partial) recovery (the so-called ‘relapsing remitting’ stage). This stage is often followed by a slowly progressive increase in disability (the stage of ‘secondary progression’). Other patients develop progressive increase in neurological disability from onset, without obvious relapses and remissions (‘primary progressive form’) [1].

Focal inflammatory demyelinating lesions that develop in eloquent areas within the CNS cause relapses. The progressive phase of MS, either secondary or primary, reflects a poorly understood insidious widespread axonal degeneration that is age-related and independent of relapses [2-4]. Currently available disease-modifying treatments, which act by modifying the inflammatory response, reduce the frequency of relapses, but are not effective in progressive MS [5-7].

Astrocytes in MS appear to be deficient in ß_2_ adrenergic receptors [8], which activate a Gs protein that associates with adenylate cyclase, leading to the conversion of ATP to cAMP, which in turn activates protein kinase A (PKA). Norepinephrine, via the stimulation of ß_2_ adrenergic receptors, tightly suppresses the expression of interferon γ-induced MHC class II molecules on cultured astrocytes [9]. We have proposed that downregulation of ß_2_ adrenergic receptors on astrocytes in MS may alter the phenotype of astrocytes into facultative immunocompetent antigen presenting cells that can initiate the inflammatory reactions leading to demyelination [10,11]. Fluoxetine activates PKA in astrocytes [12], and might thus compensate for the astrocytic ß_2_ adrenergic receptor deficiency. Based on this hypothesis we performed a pilot study in patients with relapsing remitting MS and found that a daily dose of 20 mg tended to reduce the formation of new inflammatory lesions on magnetic resonance imaging (MRI) of the brain [13].

Mechanisms proposed to be involved in the progressive axonal degeneration in MS are reduced axonal energy metabolism, axonal glutamate toxicity, and reduced cerebral blood flow [14-16]. This might also be mediated by astrocytic dysfunction, associated with reduced astrocytic ß_2_ adrenergic receptors [11]. Fluoxetine might reduce progressive axonal loss in MS, through activation of PKA, as it stimulates astrocytic glycogenolysis necessary for maintenance of sodium-dependent glutamate uptake by astrocytes, and the release of lactate, which serves as energy source for axons [17,18]. Fluoxetine also stimulates the release of the neuroprotective brain-derived neurotrophic factor (BDNF) from astrocytes [18], and may improve cerebral blood flow by dilating cerebral arterioles independent of the endothelium [19].

Two weeks of treatment with fluoxetine (first week 20 mg/day and second week 40 mg/day) significantly improved cerebral white matter N-acetylaspartate/creatine ratio in MS patients, suggesting an improvement in axonal mitochondrial energy metabolism, because N-acetylaspartate is produced by mitochondria [20].

Based on these preliminary findings we decided to perform a randomized placebo-controlled trial to assess whether fluoxetine has a neuroprotective effect in patients with progressive MS.

Secondary and primary progressive MS have a different form of symptom onset but the underlying pathophysiological mechanism leading to progressive axonal degeneration is likely the same. It is independent of relapses, and the progressive phases of each proceed at remarkably similar rates [1]. In addition the difference between the progressive types is not always clear cut and often arbitrary as subjects with primary progressive MS can also experience relapses.

## Methods/Design

### Trial design

FLUOX-PMS (FLUOXetine in Progressive Multiple Sclerosis) is a multi-center, randomized, controlled parallel group, and double-blind clinical trial conducted at different sites located in the Flemish region of Belgium, and in The Netherlands. Recruitment in Belgium and The Netherlands started in February 2012 and April 2013, respectively. In total, 120 patients with either secondary or progressive MS will be randomized to receive either two tablets of fluoxetine 20 mg or placebo of identical appearance, daily for 108 weeks. The study is approved by the local ethics committees (See Additional file 1) and at the national level by ‘Federaal Agentschap voor Geneesmiddelen en Gezondheidsproducten’ (Belgium) and the ‘Centrale Commissie Mensgebonden Onderzoek’ (Netherlands). The study is registered at the European Union Drug Regulating Authorities (Eudra-CT: 2011-003775**-**11). The study will be conducted in accordance to the Declaration of Helsinki in its currently applicable version, the guidelines of the International Conference on Harmonization of Good Clinical Practice (ICH-GCP), and the applicable Belgian and Dutch legislation. All participants are required to give written informed consent.

### Participants

Inclusion criteria for participation in the FLUOX-PMS trial comprise the diagnosis of either secondary or primary progressive MS according to the revised 2010 McDonald criteria [21], aged 25 to 65 years, a score between 3 and 6.5 included on the Expanded Disability Status Scale (EDSS) [22], and documented confirmed evidence of disease progression independent of clinical relapses over the 1 year prior to enrolment, defined as an increase of 0.5 point on the EDSS. For sexually active female patients with reproductive potential, use of reliable means of contraception is required. The main exclusion criteria include use of antidepressants. Concomitant medications that may lead to clinically significant interactions with fluoxetine (such as monoamine oxidase inhibitors that could lead to a serotonin syndrome) are not allowed. Other exclusion criteria are pregnancy or lactation, other neurologic, serious psychiatric (for example, major depression) or systemic disorders that may interfere with the assessments. Use of immunomodulatory or immunosuppressive drugs is not allowed, except for interferon beta or glatiramer acetate, as it has been shown that these drugs are ineffective in slowing down progression [5-7]. Patients using other immunosuppressive or immunomodulatory drugs can only be included if stopped at least for 2 months before randomization.

### Study mediation, randomization, and follow-up

Eligibility of patients is determined at the screening visit (week −4). At baseline visit, patients who qualify for participation in the study are randomized 1:1 using a block size of 10, to receive either fluoxetine or placebo. Study medication (verum and placebo) was purchased from Eurogenerics and stored at the department of Pharmacy of the UZ Brussel. After inclusion, patients are given a computer-generated randomization number, which is matched to a confidential treatment number by the study pharmacist of the UZ Brussel to assign patients either to fluoxetine or to placebo.

Trained study nurses visit patients at home to perform all assessments in their natural environment, avoiding the stress and efforts of going to the hospital, which may interfere with clinical assessments. They plan the brain MRI scans, provide the study medication, and will be contacted in case of adverse events, suspected relapse, or questions. Counting tablet returns at each visit will assess adherence. In case of a suspected relapse they will refer the patient to their treating neurologist. All patients are followed as usually by their treating neurologist. When there is a change in immunomodulatory treatment or antidepressants have to be started, the treating neurologist is kindly asked to notify the study team.

**Figure 1:**
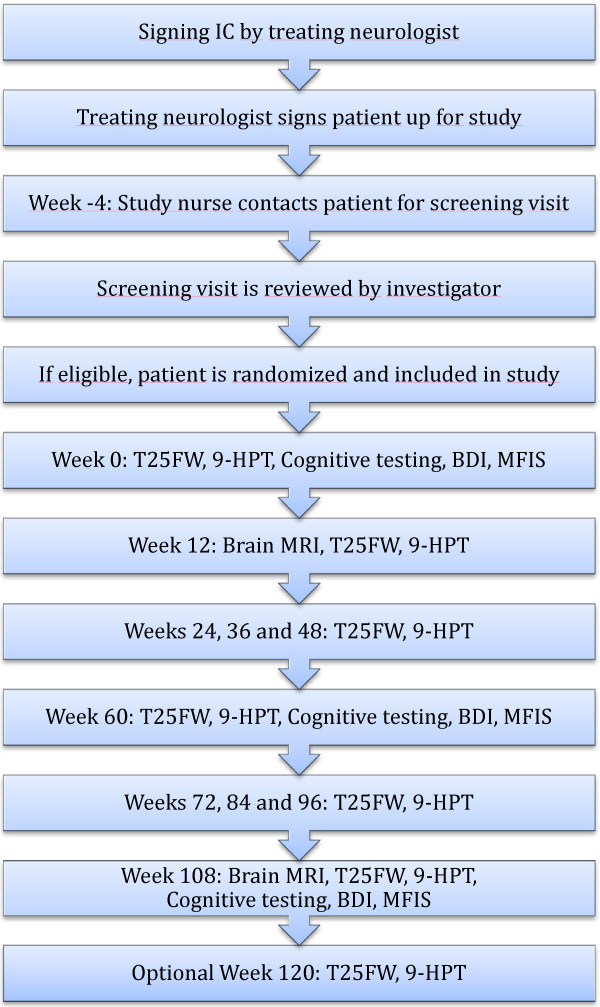
Trial flow chart (see also list of abbreviations).

Regular study visits are carried out at weeks 0, 12, 24, 36, 48, 60, 72, 84, 96, and 108. An optional follow-up visit will be offered at week 120 to confirm sustained progression appearing at week 108. If necessary, additional unscheduled visits can be performed at any time. The study flow-chart is shown in Figure 1.

### Primary outcome parameter

The primary endpoint is the time to confirmed disease progression defined as either at least a 20% increase in the timed 25-Foot Walk (T25-FW) or at least a 20% increase in the 9-Hole Peg Test (9-HPT; assessment of upper limb function). In each situation the change cannot be attributed to another etiology (for example, fever, concurrent illness, relapse), and is sustained for at least ≥12 weeks up to the end of the trial.

In the T25-FW the patient is directed to one end of a clearly marked 25-foot course and is instructed to walk 25 feet (7.26 m) as quickly as possible, but safely. The time is calculated from the instruction to start and ends when the patient has reached the 25-foot mark. The task is immediately administered again by having the patient walk back the same distance. The score for the T25-FW is the average of the two completed trials. Patients may use assistive devices when doing this task. Because mobility may be affected by factors such as spasticity and the use of different walking aids, subsequent tests between weeks 12 and 120 will be performed with the same walking aid if any, and on the same dose of anti-spasticity medication if any.

In the 9-HPT, the patient is seated at a table with a small, shallow container holding nine pegs and a wood or plastic block containing nine empty holes. On a start command when a stopwatch is started, the patient picks up the nine pegs one at a time as quickly as possible, puts them in the nine holes, and, once they are in the holes, removes them again as quickly as possible one at a time, replacing them into the shallow container. The total time to complete the task is recorded. Two consecutive trials with the dominant hand are immediately followed by two consecutive trials with the non-dominant hand. The overall 9-HPT score is the average of the four trials.

### Secondary outcome parameters

The following secondary clinical endpoints will be assessed: (1) the proportion of patients without sustained 20% increase in the T25-FW, or without 20% increase in the 9-HPT between weeks 12 and 108; (2) the proportion of patients with a stable Hauser ambulation index; (3) cognitive changes measured through a cognitive battery; and (4) fatigue.

The Hauser ambulation index is a rating scale to assess mobility by evaluating the time and degree of assistance required to walk 25 feet. Scores range from 0 (asymptomatic and fully active) to 10 (bedridden). The patient is asked to walk a marked 25-foot course as quickly and safely as possible. The examiner records the time and type of assistance (for example, cane, walker, crutches) needed.

The cognitive battery consists of the Symbol Digit Modalities Test (SDMT), California Verbal Learning Test–II (CVLT-II), and Controlled Oral Word Association Test (COWAT). The Beck depression inventory-II (BDI-II) will be used to get a view on the depression level of the patient.

Fatigue will be assessed with the Modified fatigue Impact Scale (MFIS).

Secondary surrogate endpoints comprise brain magnetic resonance imaging (MRI) data, and in some selected centers changes in the retinal nerve fiber layer thickness and macular volume between weeks 12 and 108 will be measured using optical coherence tomography (OCT).

### MRI of the brain

MRI analyses will be performed in five hospitals on a 3 tesla MRI scanner (Philips and Siemens). During each scan session, the following sequences are acquired: 3D T1 (TE, 2.93 ms; TR, 2,300 ms; TI, 900 ms; voxel size, 1.1 × 1.1 × 1.2 mm^3^; acquisition time, 5.12 min), 3D T2 (TE, 402 ms; TR, 3,200 ms; voxel size, 1 × 1 × 1 mm^3^; acquisition time, 4.43 min), 3D FLAIR (TE, 388 ms; TR, 5,000 ms; TI, 1,800 ms; voxel size, 1 × 1 × 1 mm^3^; acquisition time, 5.52 min), DTI (32 diffusion directions, b, 800 s/mm^2^; TE, 85 ms; TR, 8,500 ms; voxel size, 2 × 2 × 2 mm^3^; acquisition time, 9.48 min). Each subject will undergo two scans (at weeks 12 and 108) at the same hospital to guarantee that both scans are made under identical conditions. An automated software algorithm will be used to detect focal MS lesions. This performs an intensity-based tissue classification using a stochastic model for normal brain images and detects MS lesions as outliers that are not well explained by the model. The T1, T2, and FLAIR images will be used as input for the lesion segmentation software. Reduction in brain parenchymal fraction (BPF) is used as a normalized measure of whole-brain atrophy. BPF is measured from the 3D T1-weighted images from each time point using a fully automated method. Diffusion tensor MRI (quantified by fractional anisotropy and apparent diffusion coefficient) will be used to assess white matter tissue integrity. To this end, all DTI datasets will be aligned to a population-specific atlas. Subsequently, diffusion parameters will be evaluated statistically in every white matter voxel. All MRI parameters will be gathered and assessed by an experienced evaluator (ICOMETRIX) who is blinded to both clinical data and treatment allocation. Because it is a secondary outcome parameter, brain MRI is not compulsory for patients having difficulty to undergo this examination.

### Sample size

The sample size was estimated on the basis of a study in untreated patients with primary progressive multiple sclerosis [23]. The primary event is sustained worsening on the T25-FW or 9-HPT within 2 years using a survival analysis. At 2 years the fraction of event-free patients is estimated to be 45% in the untreated group, and 70% in the fluoxetine-treated group. Using sample size calculation for comparing two survival curves (StatemateTM, GraphPad software, San Diego, CA, USA), including 60 patients in each group has 80% power to detect an increase in survival proportion of 0.25 with a significance level (alpha) of 0.05 (two-tailed). For every patient who drops out within the first 3 months, which is the period when we expect most drop-outs, a new patient will be included until a sample size of 120 patients is reached.

### Blinding and statistical methods

Patients and the entire study staff will remain blinded throughout the complete treatment period. Treatment allocations will only be disclosed after the final database lock. Independent examiners will do MRI and OCT evaluations. All data will be statistically analyzed by an independent biometrician. Proportion of patients will be analyzed with the use of a chi-square test. A log-rank test will be applied to compare differences in time to confirmed disease progression between the two treatment groups. A Cox proportional hazard model will be used to estimate the reduction in hazard associated with treatment compared with placebo. Repeated measures analysis of covariance will be used to compare differences between the fluoxetine and placebo group in endpoints related to changes from baseline. The test level for statistical significance of differences between both treatment arms is defined as *P* = 0.05 for all tests.

## Discussion

So far, no drug has been able to slow down progressive MS. Fluoxetine is mainly indicated for the treatment of mild-to-moderate depression, but the drug has potential pleiotropic neuroprotective effects [24]. The patent of fluoxetine expired in 2001. Because it is an old drug its side-effect profile, which is in general mild, is well established.

Some aspects of the trial need clarification. First, because it may take several weeks before a steady state of fluoxetine level in blood and brain is achieved [25], progression is measured from the second visit at 12 weeks, and not from start of the trial. Second, the choice of the dose of fluoxetine 40 mg is based on clinical practice indicating that dosages of 20 to 40 mg are effective in neuropsychiatric disorders. Third, the trial lasts for 2 years, because observing progression for 2 years is more meaningful than for 1 year [23]. Fourth, we decided not to use the EDSS as primary outcome measure because study nurses assess the patients in our study and the EDSS is a rather insensitive measure to assess progression. Fifth, we use cutoffs of 20% for both T25-FW and 9-HPT, because this has a better signal-to-noise ratio than lower values (for example, 15%) and is therefore preferable for the assessment of disease progression [26]. In a group of 161 primary progressive multiple sclerosis patients, Bosma and colleagues found that progression as measured with the objective outcome measures T25-FW or 9-HPT was 56% at 2 years. Combining T25-FW or 9-HPT or EDSS resulted in a slightly higher event rate of 63%. Sixth, we do not exclude patients on first-line immunomodulatory drugs (interferons and glatiramer acetate) since they have shown in clinical trials to be inefficacious in progressive MS. From a methodological point of view, a design without immunomodulatory drugs would be preferable. Because many neurologists use these drugs off-label in progressive MS we feel that excluding these patients would seriously hamper patient recruitment. The main reason for exclusion during the screening phase is the use of antidepressants, which is frequently indicated in progressive MS patients.

Our trial has a unique design, which, to the best of our knowledge, has not been used before. Traditionally, MS patients participating to a clinical trial are assessed on a regular basis by neurologists and study nurses at the hospital. In this study, the trained study nurse assesses patients at their home. This avoids stress and physical effort associated with attending the hospital, and ensures that all functional tests are done in the patient’s natural environment.

## Trial status

The FLUOX-PMS trial is currently recruiting patients.

## Abbreviations

9-HPT: 9-Hole Peg Test; BDI-II: Beck Depression Inventory-II; CNS: Central nervous system; COWAT: Controlled Oral Word Association Test; CVLT-II: California Verbal Learning Test–II; EDSS: Expanded disability status scale; FLUOX-PMS: Fluoxetine in Progressive Multiple Sclerosis; ICH-GCP: International Conference on Harmonization of Good Clinical Practice; MRI: Magnetic resonance imaging; MS: Multiple sclerosis; OCT: Optical coherence tomography; PKA: Protein kinase A; SDMT: Symbol digit modalities test; T25-FWx: Timed 25-Foot Walk.

## Competing interests

The authors declare that they have no competing interests. The study sponsors have no role in the study design, data collection, data analysis, data interpretation, or writing of the report.

## Authors’ contributions

MC and JD conceptualized and designed the study. JD obtained the funding. PH is responsible for the statistical part. WV is responsible for the coordination and analysis of the MRI data. VM trained the nurses for the cognitive testing. AVW and MC trained the nurses and coordinate the study. JD, MC, JM, MD, GN, BW, DH, JD, LA, ND, EF, GL, HM, BV, LV, WV, RH, and GH are recruiting the patients. JD and MC drafted the manuscript. All authors read and approved the final manuscript.

## Supplementary Material

Additional file 1 Description of data: List of ethical committees of recruiting centers. Click here for file
