# Supplementary material for: Fluoxetine in Progressive Multiple Sclerosis (FLUOX-PMS): study protocol for a randomized controlled trial
Source: Trials. 2014 Jan 25;15:37. doi: 10.1186/1745-6215-15-37 (PMC3931920; doi:10.1186/1745-6215-15-37)
Supplement: Additional file 1 — Description of data: List of ethical committees of recruiting centers. [file 1745-6215-15-37-S1.docx]

# Additional file 1

**Ethical committees of the participating centers**

**Belgium**

UZ Brussel*

Elisabeth Ziekenhuis Campus Sijsele

AZ St. Jan Brugge

AZ Maria Middelares Gent

UZ Gent

Nationaal MS centrum Melsbroek

Stedelijk Ziekenhuis Aalst

St-Maria ziekenhuis Halle

OLV ziekenhuis Aalst

UZA Antwerpen/ Mick

H.-Hartziekenhuis Menen

MS centrum Overpelt

AZ Damiaan Oostende

AZ Sint-Jozef Turnhout

Kortrijk AZ Groeninge

Virga Jesse ziekenhuis Hasselt

Centre Neurologique et de Réadaption Fonctionelle

**The Netherlands**

Universitair Medisch Centrum Groningen

Orbis medisch centrum Sittard / Academisch MS centrum Limburg

Rijnstate Arnhem

Canisius Wilhelmina Ziekenhuis (CWZ) Nijmegen

Catharina Ziekenhuis Eindhoven

* Central ethical committee
